# Environmental enrichment decreases anxiety‐like behavior in zebrafish larvae

**DOI:** 10.1002/dev.22255

**Published:** 2022-03-20

**Authors:** Elia Gatto, Marco Dadda, Matteo Bruzzone, Enrico Chiarello, Gaia De Russi, Marco Dal Maschio, Angelo Bisazza, Tyrone Lucon‐Xiccato

**Affiliations:** ^1^ Department of Chemical Pharmaceutical and Agricultural Science University of Ferrara Ferrara Italy; ^2^ Department of Life Sciences and Biotechnology University of Ferrara Ferrara Italy; ^3^ Department of General Psychology University of Padova Padova Italy; ^4^ Padua Neuroscience Center–PNC University of Padova Padova Italy; ^5^ Department of Biomedical Sciences University of Padua Padova Italy

**Keywords:** anxiety‐like behavior, *Danio rerio*, neophobia, novel object recognition test, stimulus avoidance

## Abstract

The development of anxiety disorders is often linked to individuals’ negative experience. In many animals, development of anxiety‐like behavior is modeled by manipulating individuals’ exposure to environmental enrichment. We investigated whether environmental enrichment during early ontogenesis affects anxiety‐like behavior in larval zebrafish. Larvae were exposed from hatching to either an environment enriched with 3D‐objects of different color and shape or to a barren environment. Behavioral testing was conducted at different intervals during development (7, 14, and 21 days post‐fertilization, dpf). In a novel object exploration test, 7 dpf larvae of the two treatments displayed similar avoidance of the visual stimulus. However, at 14 and 21 dpf, larvae of the enriched environment showed less avoidance, indicating lower anxiety response. Likewise, larvae of the two treatments demonstrated comparable avoidance of a novel odor stimulus at 7 dpf, with a progressive reduction of anxiety behavior in the enriched treatment with development. In a control experiment, larvae treated before 7 dpf but tested at 14 dpf showed the effect of enrichment on anxiety, suggesting an early determination of the anxiety phenotype. This study confirms a general alteration of zebrafish anxiety‐like behavior due to a short enrichment period in first days of life.

## 1 INTRODUCTION

Environmental conditions and individuals’ experiences, especially during early development, have remarkable effects on human brain functioning, cognition, emotions, and behavior, ultimately affecting how individuals cope with the current situation (Dawson et al., 2000; Fox et al., 2010; Graziano et al., 2010). There is increasing recognition that some psychopathologies, such as anxiety disorders, are neurodevelopmental in their origins (Chorpita & Barlow, 1998; Gross & Hen, 2004; Loman & Gunnar, 2010). The mechanisms underlying developmental anxiety have been classically investigated with large use of models based on laboratory mammalian species, which display behavioral phenotypes, often referred to as anxiety‐like behavior, considered homologous to human anxiety (Ganella & Kim, 2014; Sanchez et al., 2001; Tarantino et al., 2011). In this context, one of the most powerful study systems consists in the comparison of brain and behavior of animals exposed to enriched versus barren environmental conditions (Benaroya‐Milshtein et al., 2004; Hendershott et al., 2016; Hüttenrauch et al., 2016; Sampedro‐Piquero et al., 2013). Elements of enrichment are usually objects that increase the structural complexity of the environment in which the subjects are raised (Näslund & Johnsson, 2016). Animals exposed to environmental enrichment are more tolerant to introduction of novel elements and typically show low anxiety phenotypes (Görtz et al., 2008; Hüttenrauch et al., 2016). In addition, studies on animal models have demonstrated the potential role of enrichment as treatment to reduce anxiety‐like behavior (Görtz et al., 2008; Sampedro‐Piquero et al., 2016).

In recent years, studies on anxiety‐related neurobehavioral plasticity have begun to exploit nonmammalian vertebrates such as the teleost fish (Näslund et al., 2012; Zhang et al., 2019). These animals are expected to contribute significantly to the field because of their neurobiology and methodological advantages. For instance, fish brain is characterized by extensive plasticity in response to environmental conditions (Ebbesson & Braithwaite, 2012) and potential for extended neurogenesis throughout life (Zupanc, 2006). For some species such as the zebrafish, *Danio rerio*, a range of molecular and behavioral tools are available to model anxiety disorders and other neuropathologies (Maximino et al., 2010; Stewart et al., 2012). If used at the larval stage, the zebrafish shows the added advantage to permit large, population‐level screenings of behavioral phenotypes and genotypes (Muto et al., 2005; Rihel et al., 2010), and even to visualize whole‐brain activation with single neuron resolution during behavioral tests (Cong et al., 2017). For these reasons, larval zebrafish are becoming the main model in developmental neurobiology, including for personalized psychology (Volgin et al., 2019).

Evidence of effects of environmental enrichment on zebrafish anxiety‐like behavior have been accumulating rapidly (Collymore et al., 2015; Dos Lee et al., 2019; Marcon et al., 2018; Santos et al., 2020; Zellner et al., 2011). However, in spite of the increasing use of larval zebrafish for anxiety research, all of this evidence almost exclusively concerns adult subjects. In this scenario, our work aimed to describe a larval zebrafish model for anxiety based on environmental enrichment. We raised zebrafish larvae in either enriched environment or control barren conditions and assessed their behavior at the age of 7, 14, and 21 days post‐fertilization (dpf). We used two behavioral paradigms. The first one is a standard novel object exploration test (experiment 1), in which the subject is exposed to a novel object and the tendency to avoid is used as proxy of anxiety behavior (Bruzzone et al., 2020; Dahlbom et al., 2011; Johnson & Hamilton, 2017; Kysil et al., 2017; Toms and Echevarria, 2014; Wright et al., 2006). As we used objects of various color and shape in the enrichment treatment, the outcome of experiment 1 could potentially be a consequence of visual enrichment on visual perception or the result of the similarity between the objects used in the enrichment treatment and the testing stimulus. Therefore, in experiment 2, we assessed larvae’ anxiety state with a completely different class of stimuli, new odors, using the novel odor exploration test (Lucon‐Xiccato et al., 2020). The former experiments did not detect an effect of enrichment in the younger groups of larvae (7 dpf) and failed to clarify whether this was due to the age of the subjects or to the shorter duration of enrichment treatment. To disentangle the two possibilities, we performed a third experiment. Two groups of larvae were exposed to the enrichment and tested at the age of 14 dpf, with the novel object exploration test. One group was exposed to the enrichment as the 7 dpf group of experiments 1 and 2, whereas the second group of larvae was exposed to an enrichment treatment with the same length (3 days) but just before the test (between 11 and 13 dpf).

## 2 MATERIALS AND METHODS

### 2.1 Ethical statement

The experiments of this study adhere to the current legislation of our country (Italy, D.L. 4 Marzo 2014, n. 26) and were approved by the Ethical Committee of University of Padova (protocol no. 61/2018) and University of Ferrara (protocol no. TLX‐2019‐1).

### 2.2 Subjects and housing condition

We tested 310 zebrafish larvae overall, 120 larvae in experiment 1, 120 larvae in experiment 2, and 70 larvae in experiment 3. Larvae were obtained by natural spawning from wild‐type breeders (16 breeding pairs) from an outbred stock. Breeders were maintained in 150‐L glass tanks provided with natural gravel and vegetation. Each tank housed mixed sex groups of approximately 30 individuals. Water temperature was kept at 28 ± 1°C with nitrite levels <0.1 mg/L and general hardness 5−10°d. Fluorescence lamps provided illumination with a 14:10 light:dark cycle. Fish were fed three times per day with live brine shrimp nauplii (*Artemia salina*; Ocean Nutrition, USA) and commercial flakes.

Pairs of males and females were collected from maintenance tanks and moved into standard breeding aquaria (Tecniplast, Italy). They were kept overnight in the same breeding aquarium but separated by a partition. At light on in the following morning, the partition was removed to allow spawning. Eggs were collected within 3 h from spawning and moved into Petri dish filled with FishWater 1× (chemical composition for 1 L deionized H_2_O: 0.5 mM NaH_2_PO_4_⋅H_2_O, 0.5 mM Na_2_HPO_4_⋅H_2_O, 1.5 g Instant Ocean, methylene blue 0.0016 g). At the hatching (about 4 dpf), the larvae were grouped in cohorts of 30 fish and moved into small tanks (L 14 cm × W 7 cm × H 4 cm) filled with 240 ml of water for the enrichment treatment (60 mg Instant Ocean salt and 0.0016 g methylene blue per L of distillate H_2_O).

### 2.3 Environmental enrichment treatment in experiments 1 and 2

**FIGURE 1:**
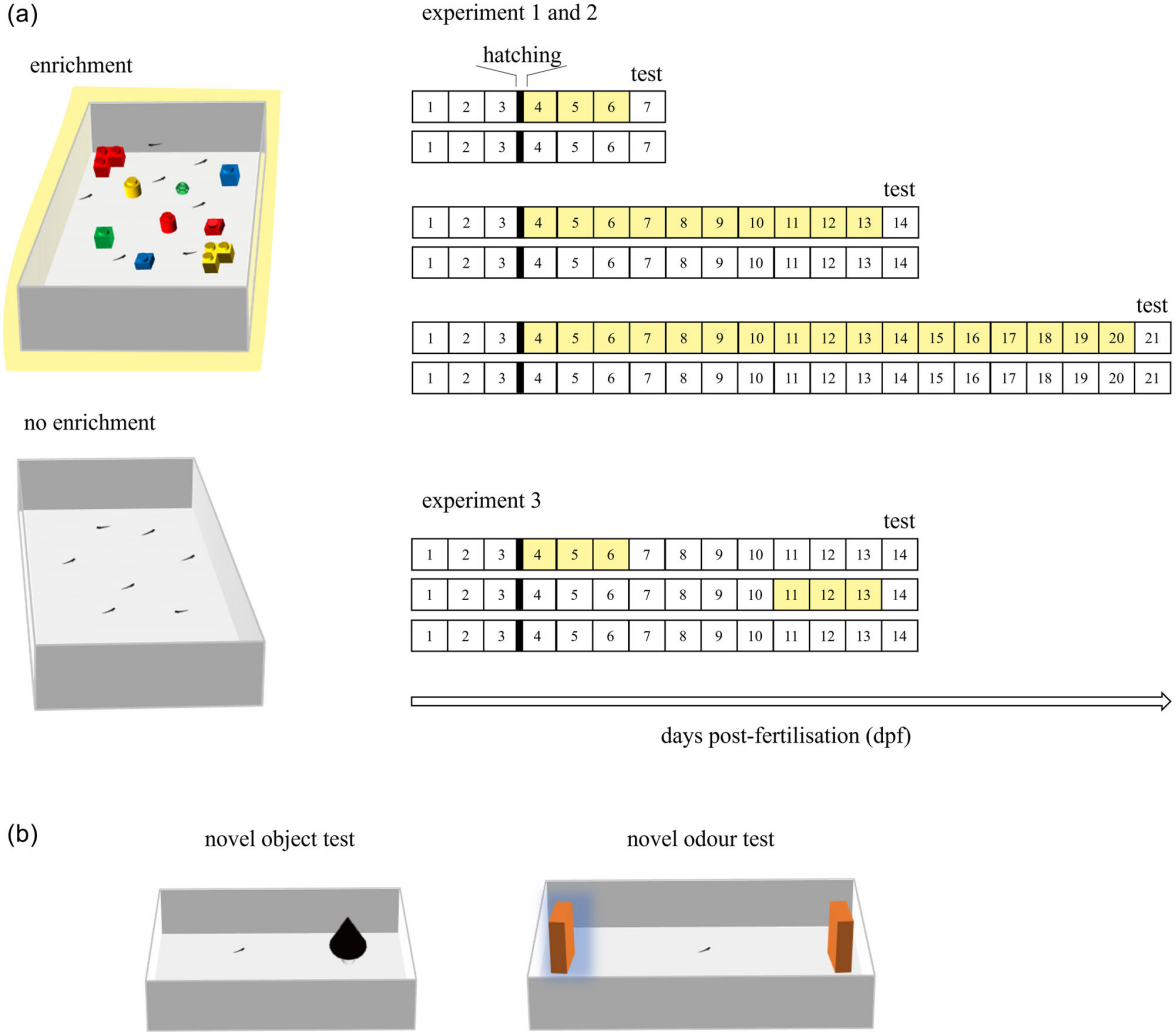
Schematic diagram of the experimental design. (a) Enrichment treatments. Zebrafish larvae were randomly assigned to either “enrichment” or “no enrichment” treatment. As enrichment, Lego® bricks of various color and shape were provided to increase environmental complexity. Larvae were individually tested at different age according to the experimental schedule. Experiment 1 aimed at investigating the subjects’ reaction to a novel visual stimulus at three different ages: 7, 14, and 21 dpf. Experiment 2 aimed at investigating the subjects’ reaction at 7, 14, and 21 dpf to a novel odor. Experiment 3 aimed at investigating the effect of environmental enrichment duration and timing on subject's reaction to a novel object stimulus in 14‐dpf larvae exposed either from 4 to 6 dpf or from 11 to 13 dpf or not exposed to enrichment. (b) Behavioural apparatuses. Novel object test of experiments 1 and 3 (left): larvae were free to interact with a novel object (i.e., a black cone place over a white pedestal). Novel odor test of experiment 2 (right): larvae were free to explore undisturbed the two stimuli. One stimulus was soaked with a solution of FishWater and olfactory cue (Benzaldehyde), while the other had no olfactory cue and was used as control

For experiments 1 and 2, we collected larvae from 12 breeding pairs. After hatching (4 dpf), each brood was randomly split in half, and each of the resulting groups was assigned to one of the two treatments (“enrichment” or “no enrichment”). We set up five tanks for each treatment condition. The enrichment treatment simulated development in an enriched environment with various structurally complex objects. As enrichment, 9 Lego® bricks of various colors (“Red”, hex code E51E26, Red‐Green‐Blue scale: 229, 30, 38; “Green”, hex code 00A8AF, RGB: 0, 168, 79; “Blue”, hex code 006CB7, RGB: 0, 108, 183; “Yellow”, hex code F7D112, RGB: 247, 209, 18) and various shapes (L‐shape, brick, cylinder, flower‐shape) were inserted in each treatment tank (Figure 1(a)). Lego bricks have been used in previous studies in fish (Bruzzone et al., 2020; Dahlbom et al., 2011) and other species (e.g., Bettis & Jacobs, 2012). They provide an excellent set of stimuli available to most laboratories, which is expected to favor replicability of the paradigm. The second treatment (“no enrichment”) consisted in keeping the larvae into an empty tank, with no exposure to stimuli. In both treatments, surplus food and fecal material were removed daily with a Pasteur pipette and 30% of water was replaced with fresh solution. In experiment 2, to habituate subjects to the odor of the sponge used to deliver olfactory stimulus during the behavioral test (see below), 10 sponges were soaked in each liter of the new water during the night before the change. These sponges were removed before adding the water to the fish tanks to avoid any visual cueing. During water changing, the position of the stimuli was randomly altered.

Larvae were fed with dry food (particle size: 50–100 μm) twice per day and maintained under treatment until the start of the behavioral experiments. When larvae reached the age of 6, 13, and 20 dpf (i.e., the day before the behavioral testing), objects were removed from the tanks to allow collecting the subjects for the behavioral experiments. The following day, we inserted new sets of objects in the tanks to continue the treatment for the larvae that were not tested in that time point. The new sets contained 9 Lego® bricks (as the previous set) with two randomly chosen objects, which were different in shape. To match the manipulation done for the enriched treatment, we simulated the same procedure in the “no enrichment” group. The behavioral testing was conducted in subjects with different developmental stage: 7, 14, and 21 dpf. The testing ages were selected to encompass the entire larval period of this species. After the age of 21 dpf, zebrafish undergo critical physiological and morphological changes such as transition from skin to gill respiration (Hale, 2014) and beginning of juvenile gonadal development (Orban et al., 2009). Behavioral traits also incur to significant changes at this age (Valente et al., 2012). As subjects were randomly assigned to the treatments at 4 dpf and kept in their respective tanks until the behavioral testing, the treatments (enriched or barren) lasted 3, 10, and 17 days for larvae tested at 7, 14, and 21 dpf, respectively. Twenty subjects of each age were tested per each condition of each experiment.

### 2.4 Experiment 1: Novel object test

For the behavioral testing, subjects were individually collected using a Pasteur pipette and moved into the experimental apparatuses. The apparatuses were made of white plastic (L 7 cm × H 4 cm × W 5 cm) and filled with 90 ml of FishWater (Figure 1(b)). The object stimulus was a black cone with base diameter 0.7 cm and 1 cm height, roughly corresponding to 213% the average length of a subject (average size 3.29 ± 0.31 mm). The cone was placed over a white pedestal (H 1.3 cm) and inserted in the center of one half of the tank. In the different subjects, the position of the stimulus was left/right counterbalanced between the two halves of the tank. Each subject was left undisturbed and free to interact with the object for 10 min. A camera (Canon LEGRIA HF R38) placed above the apparatus at a distance of 90 cm was used to record the behavior of the subject under ambient illumination.

To score subjects behavior from the video recordings, we implemented an offline tracking python‐script based on the “opencv” library. The tracking was computed after background subtraction and binarization of the image, resulting in the subject as a dark object compared with the background. For each video frame, the *xy* coordinates of the subject were extracted and stored by the tracking software. A second custom written Python script based on the “pandas” (McKinney, 2010) and “opencv” libraries extracted positional information of the subject related to a reference image of the experimental apparatus. The script elaborated the image of the testing apparatus to identify two circular sectors (diameter 2 cm). One of the sectors contained the stimulus object, whereas the other sector was empty and placed in a specular position in the apparatus. Thereafter, the script calculated the time spent in each sector, the number of approaches (i.e., entering) to the sectors, and the swimming velocity in each sector. Subjects moving less than 5% of the entire test duration were discarded from the following analysis as that constant immobility could be due to health issues (*n* = 4). This threshold was calculated by the Python script as time spent moving/total testing time.

### 2.5 Experiment 2: Novel odor test

The novel odor test was performed following the paradigm recently devised for zebrafish (Lucon‐Xiccato et al., 2020). The protocol was designed as similar as possible to that of experiment 1 to ensure comparable results (Figure 1(b)). For the testing, subjects were individually transferred via a Pasteur pipette to the experimental apparatuses made of white plastic (15 cm × 5 cm × 5 cm) and filled with 120 ml of FishWater. The apparatus of the novel odor test was significantly longer compared with that of the novel object test because this prevented the olfactory stimulus to spread too widely in the apparatus (Lucon‐Xiccato et al., 2020). The experimental apparatuses were kept inside a high‐edged container to avoid disturbances and were lit by a LED strip from above. Larvae were free to swim undisturbed for a habituation period of 30 min. At the end of this period, a cube of cellulose sponge (side 1 cm) mounted on a 2.5 × 2.5 cm glass base was inserted at each side of the tank. One of the sponges was soaked with a solution of FishWater and olfactory cue (1%). The olfactory cue used was benzaldehyde (Sigma–Aldrich), which is typically used as stimulus in animal behavioral experiments (Lyons & Roman, 2009; Terral et al., 2019). Its position was randomized across subjects between the shorter sides of the tank. The other sponge had no olfactory cue and was used as control. This setting with two sponges allowed to analyze reaction to the novel odor without the interference of the visual perception of the novel object (the sponge).

A Canon LEGRIA HF R38 camera was placed above the apparatus at a distance of 35 cm, allowing to record the behavior of the subjects. The behavior was recorded for 10 min. The videos were analyzed through BORIS software (Behavioral Observation Research Interactive Software; University of Torino, Torino, Italy). The experimental tanks were virtually divided in length into three equivalent sections. These choice areas were larger compared with that used in experiment 1 because of the need to contain the area of the apparatus in which the odor spread. BORIS allowed to calculate the time spent by the subject in each virtual sector of the apparatus. Subjects that visit both sections less than 5% of the entire test duration were discarded (*n* = 21).

### 2.6 Experiment 3: Effect of environmental enrichment duration and timing

To evaluate the relative effects of enrichment duration and timing, we performed a third experiment with a modified treatment. Larvae collected from four breeding pairs were randomly assigned to three different treatments (Figure 1(a)). Two groups were exposed to the environmental enrichment for 3 days but with different timing, either from 4 to 6 dpf or from 11 to 13 dpf. As in previous experiments, a third, control group was not exposed to enrichment (no enrichment group). Remaining details for the treatment were as described for experiments 1 and 2. The three groups of larvae were then tested at 14 dpf with the novel object exploration test, following the same protocol described for experiment 1 (Figure 1(b)).

### 2.7 Statistical analysis

Statistical analysis was performed in RStudio version 1.2.5019 (RStudio Team, 2019; RStudio: Integrated Development for R. RStudio, Inc., Boston, MA URL http://www.rstudio.com). Statistical tests were two‐sided and the threshold for significance was set at *p* = .05.

In experiment 1, subject's behavior was analyzed in three steps. The first step focused on the time spent in the sector containing the stimulus compared with the time spent in the empty sector, which indicated attraction toward the stimulus. To obtain a single dependent variable that included this information, percentage of time spent in the stimulus’ sector was calculated as time spent in the stimulus sector/(time spent in the stimulus sector + time spent in the empty sector) × 100. This percentage calculation also produced a dependent variable that can be compared between fish spending a different amount of time the choice sectors versus the empty sector of the apparatus. A two‐way ANCOVA was performed with treatment as fixed factor. Age was fitted as covariate to consider the continuous and directional nature of subjects’ age and to investigate increasing or decreasing trends in the dependent variables. The percentage was arcsine‐squared‐root transformed before the analysis to meet the homoscedasticity assumptions of the ANCOVA. To deal with a large variation across subjects in time spent in the two sectors (range 29–217 s), the dependent variable was also weighted to the overall time of subject spent in both sectors (time spent in the object sector + time spent in the empty sector). Weighting reduced binomial error that may cause high uncertainty in estimating the real preference of subjects that spent a low amount of time in the two choice sectors (Astivia & Zumbo, 2019; Carroll & Ruppert, 2017; Maronna et al., 2019). When required, pairwise post‐hoc weighted *t*‐tests were used to investigate significant effects and interaction involving the treatment term. In the second step of experiment 1 analysis, the tendency to approach the novel object was investigated with an ANCOVA model as described above. The dependent variable was the percentage of approaches toward the novel object compared with the empty sector. To deal with a large variation across subjects in the number of approaches toward the two sectors (range 5–64), the dependent variable was weighted to the overall number of approaches toward both sectors (number of approaches toward the novel object + number of approaches toward the empty sector). In the third step of experiment 1 analysis, a linear mixed‐effects model (LMM; “lme” function from the “nlme” R package) was used to investigate difference on the swimming velocity of the subject in each sector. This model was fitted with a repeated measures variable consisting in two observations per subject: the average velocity in the object sector and the average velocity in the empty sector. We used this approach because it was not possible to calculate a percentage index as for the other dependent variables. The velocity was log‐transformed before the analysis. The model was fitted with sector and treatment as fixed effects, age was defined as covariate, and subjects ID as random effect. Post‐hoc weighted *t*‐tests were used to investigate significant effect of interaction.

Data from experiment 2 was analyzed by mean of ANCOVA performed on the percentage of time spent in the sector with the odor stimulus versus the time spent in the sector with not odor stimulus. Treatment was fitted in the model as fixed factor, age as covariate, and the dependent variable was weighted to the overall time of subject spent in both sections. Visual inspection of model residuals revealed three outliers, and they were discarded from the final dataset. Post‐hoc tests were used to investigate significant effect of interaction. A final analysis was conducted by pooling the data of experiment 1 and experiment 2 (percentage of time spent in the sector with the stimulus), fitting age, treatment, and experiment as fixed effects. In experiment 2, we also conducted an analysis on the discarded subjects, that is, those that did not show a choice between the odor stimulus and the control stimulus. This was done because this absence of choice might be also related to anxiety. We first used a chi‐square test to compare the occurrence of nonchoosing subjects of experiment 2 with that observed in experiment 1. Thereafter, we analyzed the effects of age and treatment on the likelihood of observing nonchoosing subjects in experiment 2 using a generalized linear model with binomial error distribution.

The statistical approach described for experiment 1 was also applied for analyzing the effects of enrichment duration and timing in experiment 3. The percentage of time in the sector of the novel object (weighted to the overall time in the choice areas) and the percentage of inspections towards the novel object (weighted to the total number of approaches) were analyzed using an ANCOVA model with treatment as a fixed factor. The log‐transformed swimming velocity was analyzed using an LMM fitted with treatment as fixed effect, sector as fixed effect for the repeated measures, and ID as random factor. Post‐hoc weighted *t*‐tests were used to investigate the significant effects of main factors and of interactions.

## 3 RESULTS

### 3.1 Experiment 1: Response to novel object

**FIGURE 2:**
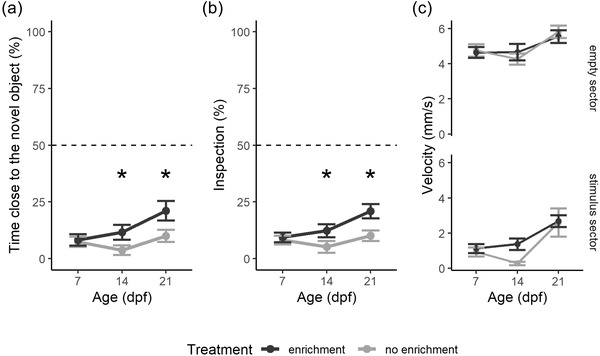
Neophobic response to novel object in 7‐, 14‐, and 21‐dpf larvae (experiment 1). (a) Percentage of time spent in the sector with the novel stimulus. (b) Percentage of inspections in the sector with the novel stimulus. (c) Swimming velocity in each sector. Subjects were divided for “enrichment” (dark gray) and “no enrichment” (light gray) treatment. Data points represented the mean ± standard error. Dotted lines represented the expected chance percentage (50%)

Analysis on the percentage of time spent in the sector with the novel object revealed a significant effect of age (ANOVA: *F*_1,112_ = 5.981, *p* = .016; Figure 2(a)), a significant effect of treatment (*F*_1,112_ = 7.880, *p* = .006), and a significant age × treatment interaction (*F*_1,112_ = 6.662, *p* = .011). Post‐hoc analysis indicated that larvae of the “enrichment treatment” group spent more time close to the novel object at the age of 14 dpf (*F*_1,37_ = 4.459, *p* = .042) and at the age of 21 dpf (*F*_1,36_ = 7.157, *p* = .011), but not at the age of 7 dpf (*F*_1,37 _=  0.127, *p* = .724).

Analysis on the percentage of inspections in the sector with the novel object revealed a significant effect of age (*F*_1,112_  =  9.143, *p* = .003; Figure 2(b)), a significant effect of treatment (*F*_1,112_  =  14.502, *p* < .001), and a significant age × treatment interaction (*F*_1,112_ = 5.134, *p* = .025). Post‐hoc analysis indicated that larvae exposed to enrichment treatment with objects performed more inspections to the novel object at the age of 14 dpf (*F*_1,37_  =  7.914, *p* = .008) and at the age of 21 dpf (*F*_1,36_  =  9.169, *p* = .005), but not at the age of 7 dpf (*F*_1,37 _=  0.037, *p* = .848).

When considering the velocity, the repeated measures ANOVA revealed a significant difference between the sector with the novel object and the empty sector (*F*_1,112 _=  498.919, *p*  <  .001; Figure 2(c)), a significant difference among ages (*F*_1,112_  = 14.730, *p* < .001), a significant effect of treatment (*F*_1,112_  =  4.284, *p* =  .041), a significant age × sector interaction (*F*_1,112 _= 8.556, *p*  =  .004), and significant treatment × sector interaction (*F*_1,112 _= 10.358, *p*  =  .002), but not significant three‐ways interaction (*F*_1,112 _= 0.604, *p* = .439). The latter significant interaction involving treatment was further analyzed with post‐hoc models divided per sector. When inside the sector with the object, subjects of the enrichment treatment swam faster compared with the subjects of the noenrichment treatment (*F*_1,114_  = 6.862, *p*  = .010). Conversely, in the empty sector, there was no difference in velocity between subjects of the two treatments (*F*_1,112_  =  0.007, *p*  = .933).

### 3.2 Experiment 2: Response to novel odor

**FIGURE 3:**
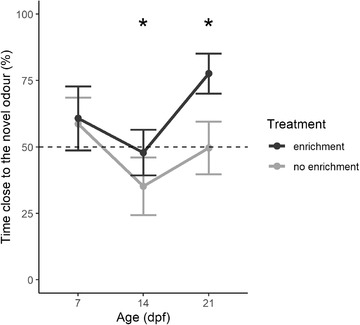
Neophobic response to novel odor in 7, 14, and 21 dpf (experiment 2). Percentage of time spent in the sector with the novel stimulus. Subjects were divided for “enrichment” (dark gray) and “no enrichment” (light gray) treatment. Data points represented the mean ± standard error. Dotted lines represented the expected chance percentage (50%)

In the ANOVA on the percentage of time spent close to the novel odor, there were no significant main effects (*p*s > .1). However, the interaction age × treatment was significant (*F*_1,91_  =  5.400, *p*  =  .022; Figure 3). Post‐hoc analysis showed that larvae of the enrichment treatment spent more time close to the novel odor compared with the larvae of the no‐object treatment at the age of 14 dpf (*F*_1,30_  =  4.188, *p*  =  .049), and at the age of 21 dpf (*F*_1,35_  =  4.401, *p* = .043), but not at the age of 7 dpf (*F*_1,24_  =  2.922, *p*  =  .100).

The analysis of the larvae that did not choose between the two sectors of the apparatus (21 out of 120 subjects) indicated a higher occurrence compared with experiment 1 (4 out of 120 subjects; chi‐squared test: *X* ^2^_1_ = 12.904. *p* < .001). The generalized linear model revealed that the likelihood of observing subjects that did not choose between the stimuli in experiment 2 decreased with age (7 dpf: 14 out of 40 subjects; 14 dpf: four out of 40 subjects; 21 dpf: three out of 40 subjects; *X* ^2^_2_ = 12.356, *p* = .002). There was no main effect of the enrichment treatment on the occurrence of nonchoosing larvae (*X* ^2^_1_ = 1.630, *p* = .202).

### 3.3 Pooled data analysis

The analysis on the pooled data of experiment 1 and experiment 2 revealed a significant main effect of age (ANOVA: *F*_1,203  _=  6.282, *p* = .013), experiment (*F*_1,203_  =  45.157, *p* < .001), and treatment (*F*_1,203_  =  5.565, *p* = .019) on the percentage of time spent in the sector with the novel stimulus. There also was a significant age × treatment interaction (*F*_1,203_  =  9.944, *p*  =  .002), confirming the findings of the prior analysis on the developmental effect of enrichment on anxiety‐like behavior. Last, the three‐ways interaction was not significant (*F*_1,203_ = 1.665, *p*  = .198), indicating a similar trend of results across the two experiments. No other interactions were significant (*p*s > .80).

### 3.4 Experiment 3: Effect of environmental enrichment duration and timing

**FIGURE 4:**
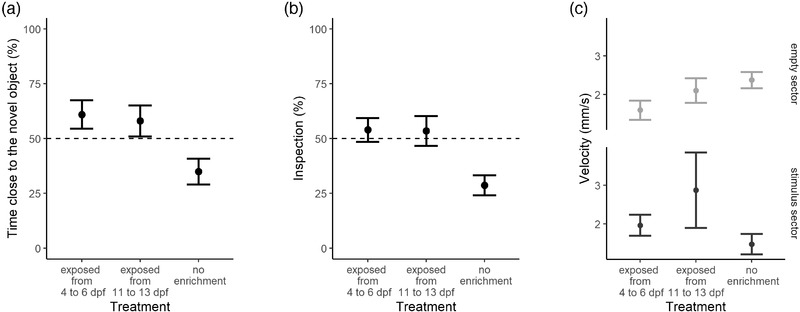
Neophobic response to novel object in 14‐dpf larvae of three treatments of experiment 3: exposed to objects from 4 to 6 dpf, exposed from 11 to 13 dpf, ‘no enrichment’ group. (a) Percentage of time spent in the sector with the novel stimulus. (b) Percentage of inspections in the sector with the novel stimulus. (c) Swimming velocity divided for the empty sector (light gray) and the sector contained the novel object (dark gray). Data points represented the mean ± standard error. Dotted lines represented the expected chance percentage (50%)

Analysis of the percentage of time spent in the sector with the novel object revealed a significant effect of treatment (*F*_2,67_  =  6.210, *p*  = .003; Figure 4(a)). Post‐hoc analysis indicated that larvae of the “no enrichment” treatment spent less time close to the novel object compared with the larvae exposed to the enrichment from 4 to 6 dpf (*F*_1,48_  =  10.366, *p* = .002) and from 11 to 13 dpf (*F*_1,48_  =  7.190, *p*  = .010). There was no difference between the larvae of the two enriched treatments (*F*_1,38 _=  0.333, *p*  = .568).

Analysis of the percentage of inspections in the sector with the novel object revealed a significant effect of treatment (*F*_2,67_  =  6.402, *p*  =  .003; Figure 4(b)). Post‐hoc analysis indicated that larvae of the “no enrichment” group performed less inspections towards the novel object compared with both the enriched treatment groups (enrichment between 4 and 6 dpf: *F*_1,48_  =  11.355, *p*  =  .001; enrichment between 11 and 13 dpf: *F*_1,48_  =  6.472, *p*  = .014). There was no difference between larvae of the two enriched treatments (*F*_1,38 _=  0.289, *p*  = .594).

When considering swimming velocity, the repeated measures ANOVA revealed a significant treatment × sector interaction (*F*_2,67 _= 6.859, *p*  = .002; Figure 4(c)). This significant interaction was further analyzed with post‐hoc models divided per sector. When inside the sector with the object, there was a significant effect of treatment on swimming velocity (*F*_2,67_  = 3.224, *p*  =  .046). This latter effect was mainly due to the higher swimming velocity showed by larvae exposed from 11 to 13 dpf compared with the “no enrichment” group (*F*_1,38_  =  5.182, *p*  =  .027), while the other pairwise comparisons did not result significant (*p*s > .099). Conversely, in the empty sector, there was no difference in swimming velocity among subjects from three treatments (*F*_2,67_  =  2.873, *p*  =  .064). No other factors were significant in the main model (*p*s > 4.15).

## 4 DISCUSSION

Results of experiment 1 indicated that zebrafish larvae raised in an enriched environment developed reduced anxiety‐like behavior compared with the larvae raised in a barren environment. This effect was visible in the main putative anxiety measures that we collected in the novel object test, that is, time spent close to the novel object and number of approaches to the novel object. Attraction toward novelty is typically considered a measure of boldness, risk taking behavior, and reduced anxiety behavior in fish and other animals (Belzung & Le Pape, 1994; Brown et al., 2007; Hamilton et al., 2021; Sundström et al., 2004). In our experiment 1, larvae of the enriched environment spent more time close to the novel object and were more prone to approach it compared with larvae of the nonenriched treatment.

Analysis of a secondary variable in experiment 1 indicated that larvae of the enrichment treatment were swimming faster when in proximity of the novel object compared with the larvae of the noenrichment treatment. This difference in swimming speed may be related to anxiety because in several studies, swimming speed negatively correlated with anxiety‐like behavior in zebrafish (Kacprzak et al., 2017; Stewart et al., 2012; Tran et al., 2016). However, other studies have suggested different relationships (Bencan et al., 2009; Cachat et al., 2010; Lau et al., 2011) and the possibility that some behavioral changes are due to maladaptive alterations in response to non‐natural enrichment treatments (Zhang et al., 2019). Therefore, it is not possible to unequivocally exclude the role of parameters other than anxiety on swimming speed.

One may argue that the features of the visual stimulus (such as size, color, and complexity) might affect the results of experiment 1 (Blaser & Heyser, 2015). To date, there is limited available knowledge on these effects in fish and it would be interesting to replicate our experiment with different stimuli (i.e., objects with different size or color). In addition, the effect observed in experiment 1 could be due to the similarity between the objects used in the enrichment treatment and the testing stimulus or to a specific effect of the enrichment treatment on visual perception system (Dolivo & Taborsky, 2017). To address these issues, in experiment 2, testing was performed with a completely different class of stimuli, new odors. The results of experiment 2 resembled those of experiment 1: larvae of the enrichment treatment developed reduced novelty avoidance toward the olfactory stimulus, and therefore reduced anxiety‐like behavior (Lucon‐Xiccato et al., 2020). The developmental effect on response to novelty was not limited to new stimuli of the same class as those used during the enrichment treatment (3D visual objects), suggesting that larvae of the two treatments developed a generalized difference in their response to novelty. Notably, we also know that zebrafish’ score in the odor exploration test positively correlates with scores in other anxiety tests involving a new environment (Lucon‐Xiccato et al., 2020). It is possible to conclude that the enrichment treatment generally altered how zebrafish interact with the environment, causing a decrease in anxiety response irrespectively to the stimulus and situation. This finding strengthened the validity of a zebrafish model for experientially driven anxiety based on enrichment treatment. In addition, it reflects findings of a general correlation between different measures of anxiety in humans (Adams & Creamer, 1974) as well as in various other animal species (Brown et al., 2007; Williams et al., 2012).

The effect of enrichment on zebrafish anxiety‐like behavior was mostly evident from the age of 14 dpf onward. This was true for both the novel object and the novel odor test. At least three biological phenomena might contribute to this age effect. First, the critical developmental period for responding to the enrichment treatment in the zebrafish brain might occur after 7 dpf (but see: Näslund et al., 2012). Second, the enrichment treatment might require a certain length to trigger the phenotypic plasticity, and in the 7‐dpf larvae, the length of the treatment (3 days) might not be long enough to produce the phenotypic change. Last, the anxiety phenotype of larval zebrafish is difficult to measure reliably at the age of 7 dpf. Indeed, it may go through normal developmental processes, as observed in zebrafish for social behavior (Buske & Gerlai, 2011; Engeszer et al., 2007; Mahabir et al., 2013) and dopaminergic and serotoninergic systems (Buske & Gerlai, 2012), and in other teleost species for anxiety‐related behaviors (Lucon‐Xiccato, Conti et al., 2020; Polverino et al., 2016). In this scenario, our experiment 3 demonstrated that a 3‐days enrichment treatment (administered between 11 and 13 dpf) is sufficient to immediately trigger plasticity in anxiety‐like behavior in 14 dpf larvae, thus rejecting the hypothesis that the age differences in experiments 1 and 2 were the consequence of treatment length. Moreover, larvae exposed to the enrichment between 4 dpf and 6 dpf developed the expected anxiety phenotype, which was however measurable later on, at the age of 14 dpf. Therefore, only the third explanation provided above seems to fit our findings. Further support for this explanation is provided by the analysis of the subjects that avoided both stimuli in experiment 2 (i.e., the sponge soaked with the odor and the control sponge), spending more than 95% of the testing time in the center of the apparatus. This response, that was likely related to some form of anxiety, significantly changed during ontogenesis. Visual comparison of the results of experiment 2 (Figure 2) and experiment 3 (Figure 4), highlights a difference in the average response between the subjects of the two similar experiments; in experiment 3, larvae show increased time close to the novel objects, increased number of inspections toward the novel object, and decreased swim velocity. Because experiment 3 was conducted as a follow‐up, and we used an outbred strain, this difference might be due to the use of different breeders to obtain the larvae. In zebrafish, large individual and genetic variation has indeed been observed for this type of behavioral traits (Dugatkin et al., 2005; Toms and Echevarria, 2014; Tran et al., 2016). Alternatively, this effect might be due to small, undetected variations in the treatment conditions or to seasonal variations in physiology and behavior (Jobling, 1987; Kneis & Siegmund, 1976; Ritcher et al., 1987; Sandström, 1983). On the one hand, this evidence indicates that the effect of enrichment is highly replicable and robust to average shifts in behavior. On the other hand, such wide variability in average behavior suggests that caution and appropriate control groups should be adopted when exploiting the novel object exploration test as a research model in zebrafish.

Research on zebrafish has gained considerable evidence on the fact that developmental exposure to various treatments can induce phenotypes with heightened anxiety‐like behavior (Baiamonte et al., 2016; Fulcher et al., 2017). This has permitted the gain of invaluable information on the molecular mechanisms of anxiety (Kacprzak et al., 2017; Lopez‐Luna et al., 2017; Parker et al., 2014). In some cases, even preclinical drug screenings have been conducted with zebrafish larvae (Maximino et al., 2014). The enrichment effect that we discovered could similarly lead to the development of zebrafish larvae models for anxiety pathology research, drug discovery, and neural plasticity. The advantages of such model rely on the extremely simple treatment, which is based on exposure to objects in the rearing tanks, and the quick effect, which is visible already at the age of 14 dpf.

Beside contributing to the understating of our brain and behavior plasticity, studies on environmental enrichment have been also relevant for welfare of laboratory animals. In adult zebrafish, evidence suggests that enrichment might increase individual's welfare (DePasquale et al., 2019; Wilkes et al., 2012), at the point that individuals actively seek environments with enrichment (Schroeder et al., 2014). Accordingly, it is commonly recommended to provide environmental enrichment in zebrafish breeding tanks (Stevens et al., 2021). Most of the welfare research in zebrafish has been conducted in adults so far, and welfare of larvae has been not properly investigated. The present study suggests that larvae also possess the brain functions necessary to perceive and respond to environmental enrichment. This raises questions on whether suggested maintenance protocols for larvae should be modified to include environmental enrichments.

## CONFLICT OF INTEREST

The authors declare no conflict of interests

## AUTHOR CONTRIBUTIONS

E. G., M. D., M. D. M., A. G., and T. L.‐X. conceived and designed the experiments; E. G. and G. D. R. performed the experiments; M. D., M. D. M., A. B., and T. L.‐X. supervised the experiments; M. B. and E. C. developed the software; E. G. performed the formal analysis; A. B. and T. L.‐X. wrote the original draft. All authors have revised the manuscript.

## Data Availability

Data are available on request from the authors
